# Effect of MDMA-Induced Axotomy on the Dorsal Raphe Forebrain Tract in Rats: An *In Vivo* Manganese-Enhanced Magnetic Resonance Imaging Study

**DOI:** 10.1371/journal.pone.0138431

**Published:** 2015-09-17

**Authors:** Chuang-Hsin Chiu, Tiing-Yee Siow, Shao-Ju Weng, Yi-Hua Hsu, Yuahn-Sieh Huang, Kang-Wei Chang, Cheng-Yi Cheng, Kuo-Hsing Ma

**Affiliations:** 1 Graduate Institute of Medical Sciences, National Defense Medical Center, Taipei, Taiwan; 2 Department of Nuclear Medicine, Tri-Service General Hospital, National Defense Medical Center, Taipei, Taiwan; 3 Department of Medical Imaging and Intervention, Chang Gung Memorial Hospital, Chang Gung University, Kueishan, Taoyuan, Taiwan; 4 Department of Biology and Anatomy, National Defense Medical Center, Taipei, Taiwan; 5 Functional and Micro-Magnetic Resonance Imaging Center, Institute of Biomedical Sciences, Academia Sinica, Taipei, Taiwan; 6 Institute of Biomedical Sciences, Academia Sinica, Taipei, Taiwan; 7 Institute of Nuclear Energy Research, Taoyaun, Taiwan; National Institute of Radiological Sciences, JAPAN

## Abstract

3,4-Methylenedioxymethamphetamine (MDMA), also known as “Ecstasy”, is a common recreational drug of abuse. Several previous studies have attributed the central serotonergic neurotoxicity of MDMA to distal axotomy, since only fine serotonergic axons ascending from the raphe nucleus are lost without apparent damage to their cell bodies. However, this axotomy has never been visualized directly *in vivo*. The present study examined the axonal integrity of the efferent projections from the midbrain raphe nucleus after MDMA exposure using *in vivo* manganese-enhanced magnetic resonance imaging (MEMRI). Rats were injected subcutaneously six times with MDMA (5 mg/kg) or saline once daily. Eight days after the last injection, manganese ions (Mn^2+^) were injected stereotactically into the raphe nucleus, and a series of MEMRI images was acquired over a period of 38 h to monitor the evolution of Mn^2+^-induced signal enhancement across the ventral tegmental area, the medial forebrain bundle (MFB), and the striatum. The MDMA-induced loss of serotonin transporters was clearly evidenced by immunohistological staining consistent with the Mn^2+^-induced signal enhancement observed across the MFB and striatum. MEMRI successfully revealed the disruption of the serotonergic raphe-striatal projections and the variable effect of MDMA on the kinetics of Mn^2+^ accumulation in the MFB and striatum.

## Introduction

3,4-Methylenedioxymethamphetamine (MDMA), sold under the street name “Ecstasy”, is an illicit recreational drug of abuse. Numerous animal performed in recent decades have demonstrated the neurotoxic effects of MDMA on central serotonergic systems [1–3]. The long-term serotonergic damage caused by MDMA was first demonstrated in laboratory animals during the mid-1980s [3–5], when MDMA was shown to produce long-lasting decreases in the number of serotonin uptake sites and in the expression of both serotonin transporters (SERT) and serotonin biomarkers (i.e., 5-hydroxyindoleacetic acid, tryptophan, and tryptophan hydroxylase). The most compelling evidence for MDMA-induced serotonergic damage comes from immunohistological studies that showed the profound loss of fine serotonergic axon terminals throughout the forebrain [6,7]. Serotonergic axons with swollen varicosity and fragmentation were visualized in the cortex, but no aberrant morphological changes in the raphe cell bodies were identified in electron microscopic analyses [8]. These results indicated that MDMA may specifically induce distal axotomy of brain serotonergic neurons.

The dorsal raphe nucleus (DRN) consists of the midbrain raphe nuclear complex that is rostrally connected to the basal-ganglia-motor system and caudally connected to the limbic system [9–11]. The DRN typically contains 40–50% of the serotonergic neurons of the brain [12] and is thought to give rise to most of susceptible of serotonergic fibers to MDMA neurotoxicity [13]. Serotonergic fibers originating from the DRN were found to be preferentially damaged following MDMA administration, whereas those originating from the medial raphe nucleus are spared [6,14,15]. Several ascending fiber tracts from the DRN have been identified in the rat central nervous system [16]. The dorsal raphe forebrain tract is a ventral transtegmental efferent pathway coursing through the midbrain forebrain bundle (MFB) and mainly terminating within the striatum [17,18]. It comprises the most prominent anterior projections of the serotonin system from the DRN in the rat brain. There is evidence from light- and electron-microscopy autoradiography studies that ascending serotoninergic axons not only pass through but also terminate within the ventral tegmental area (VTA) [17]. These long projection pathways extend for 10–11 mm within the rat brain, which makes *in vivo* investigation of the axonal integrity difficult using traditional neuroscience techniques.

Manganese-enhanced magnetic resonance imaging (MEMRI) is an emerging neuroscience investigation technique that enables *in vivo* visualization of the antegrade connections of a multi-synaptic neuronal pathway [19–22]. The manganese ion (Mn^2+^) is a paramagnetic metal ion that shortens the T_1_ relaxation time and enhances the signal in T_1_-weighted images (T1WIs). The application of MEMRI for tract-tracing exploits the chemical properties of Mn^2+^ as a calcium ion (Ca^2+^) analogue, where the induced signal enhancement is contingent upon Ca^2+^-dependent signaling events [23,24]. When injected intracranially, Mn^2+^ enters cells via voltage-gated Ca^2+^ channels, is transported along axon trajectories to the nerve terminal, and is released into the synapse, where it is selectively taken up by the neurons that are subsequently activated in the brain circuit [25]. These features render MEMRI a powerful tool for *in vivo* investigations of functional connections in complex brain systems, as well as for visualizing dysfunction of long nerve projections [19].

The present study aimed to elucidate the integrity of fibers projecting from the raphe nucleus *in vivo* using MEMRI after repeated MDMA exposure in the rat, in order to provide direct evidence of MDMA-induced axotomy of raphe-striatal projections from the dorsal raphe forebrain tract, which has previously been observed indirectly in separate *ex vivo* histology studies. Neurotoxicity was induced by administering MDMA or saline injections were administered for six consecutive days. Mn^2+^ was then injected into the raphe nucleus, and Mn^2+^ build-up within the raphe-VTA-MFB-striatum circuit was monitored using MEMRI. The kinetics of Mn^2+^ transport was characterized using the sigmoid equation proposed by Van der Linden *et al*. [22,26]. Immunohistological staining of SERT was carried out at the end of the MEMRI experiment to confirm the MDMA-induced effect on serotonergic terminals in the striatum.

## Experimental Procedures

### Animals

Experiments were carried out using 8-week-old male Sprague-Dawley rats (BioLASCO, Taipei, Taiwan) weighing between 280 and 300 g (mean: 290 g). The rats were housed in the animal facility at Academia Sinica, Taipei, Taiwan, under well-maintained environmental conditions (12:12-h light:dark cycle and controlled humidity and temperature) with free access to laboratory chow and tap water. The experimental protocol was approved by the Institutional Animal Care and Use Committee of the National Defense Medical Center, Taipei, Taiwan.

### MDMA neurotoxicity

**Fig 1:**
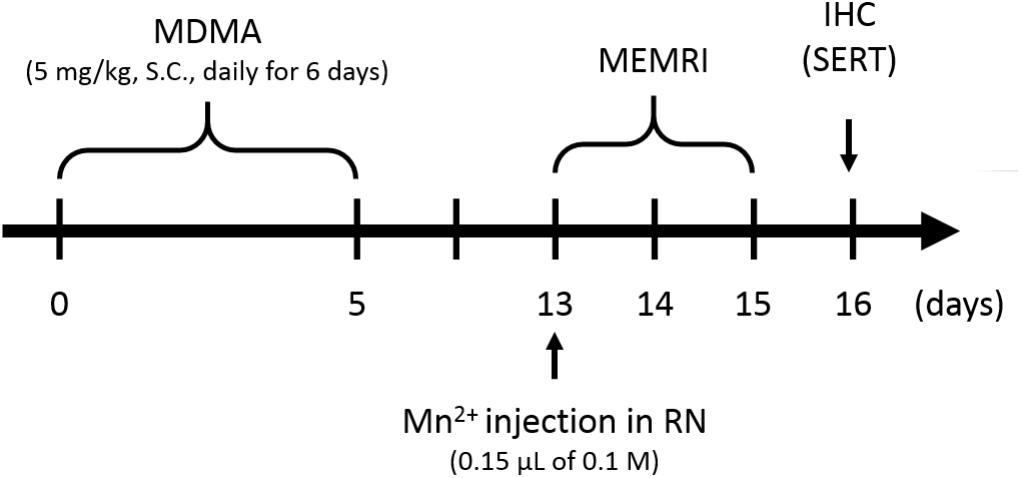
Experimental scheme for MDMA treatment, MEMRI and immunohistochemistry.

To induce MDMA neurotoxicity, five rats received daily subcutaneous (s.c.) injection of MDMA (5 mg/kg; Bureau of Investigation, Ministry of Justice, Taiwan) for six successive days. The day of MDMA treatment initiation was designated as day 0. Another group of five rats serving as controls were injected with saline using the same administration protocol. The 16-day experimental (including the subsequent MEMRI experiments) schedule is summarized in Fig 1.

### Stereotactic intracranial injection of Mn^2+^

Stereotactic intracranial injection of Mn^2+^ was performed on all rats eight days after the last MDMA or saline injection. The animals were first anesthetized with an intraperitoneal injection of chloral hydrate (Sigma-Aldrich, St. Louis, MO, USA) at a dose of 450 mg/kg. In total, 0.15 μL of 0.1 M Mn^2+^ solution (Sigma-Aldrich) was stereotactically injected into the raphe nucleus (bregma = −6.5 mm, lateral = −0.4 mm, and depth = −8.0 mm, at an angle of 43.5° from vertical) of each rat using a 33-gauge needle (Hamilton, Reno, NV, USA) in conjunction with a micro-infusion pump (Model 310; KD Scientific, Holliston, MA, USA). The total infusion time was 2 min. Stereotactic coordinates for the injection site were derived from a standard rat brain stereotactic atlas [27].

### MEMRI protocol

All MEMRI experiments were performed using a 7-T scanner (PharmaScan 70/16; Bruker, Ettlingen, Germany) equipped with an active shielding gradient (300 mT/m in 80 μs). The rats were initially anesthetized with 5% isoflurane in oxygen at a flow rate of 2L/min. When fully anesthetized, the animals were placed in a prone position and fitted with a head holder inside the magnet. The head holder is custom-designed and firmly keeps the head/coil position consistent across animal in order to minimize the signal intensity (SI) variation. In addition, all set-ups were performed by the same operator to avoid differences in positioning. Anesthesia was then maintained with 1.0−1.2% isoflurane in oxygen at a flow rate of 1L/min throughout the experiments. The rat body temperature was maintained at 37°C using a warm-water blanket. Images were acquired using a 72-mm birdcage volume coil for radiofrequency pulse transmission, and a quadrature surface coil with size of 40-mm×40-mm (Bruker, Ettlingen, Germany) was placed above the head for signal reception. To reveal the kinetics of Mn^2+^ uptake, transport, and accumulation in the brain circuits under normal and MDMA conditions, a series of coronal, sagittal, and axial T1WIs was repeatedly acquired using a two-dimensional fast and low-angle shot sequence [2D FLASH; repetition time (TR)/echo time (TE) = 200/5.0 ms, number of excitations (NEX) = 10, and flip angle = 60°] at three time intervals: 0.67 to 8 h, 22 to 28 h, and 34 to 38 h after injecting Mn^2+^ into the raphe nucleus. Eleven slices were obtained for each orientation. The scan time for one T1WI was about 9 min. To clearly locate the distribution of Mn^2+^-induced signal enhancements in the brain, T2-weighted images (T2WIs) using “rapid acquisition with relaxation enhancement (RARE)” sequence [TR/TE = 5000/70 ms, echo-train-length = 8, NEX = 2] before 2D FLASH T1WIs. All images were obtained using a field of view of 2.56 cm × 2.56 cm, a slice thickness of 1 mm, and an acquisition matrix of 256 × 128 matrix that was zero-filled to 256 × 256.

### Data processing

**Fig 2:**
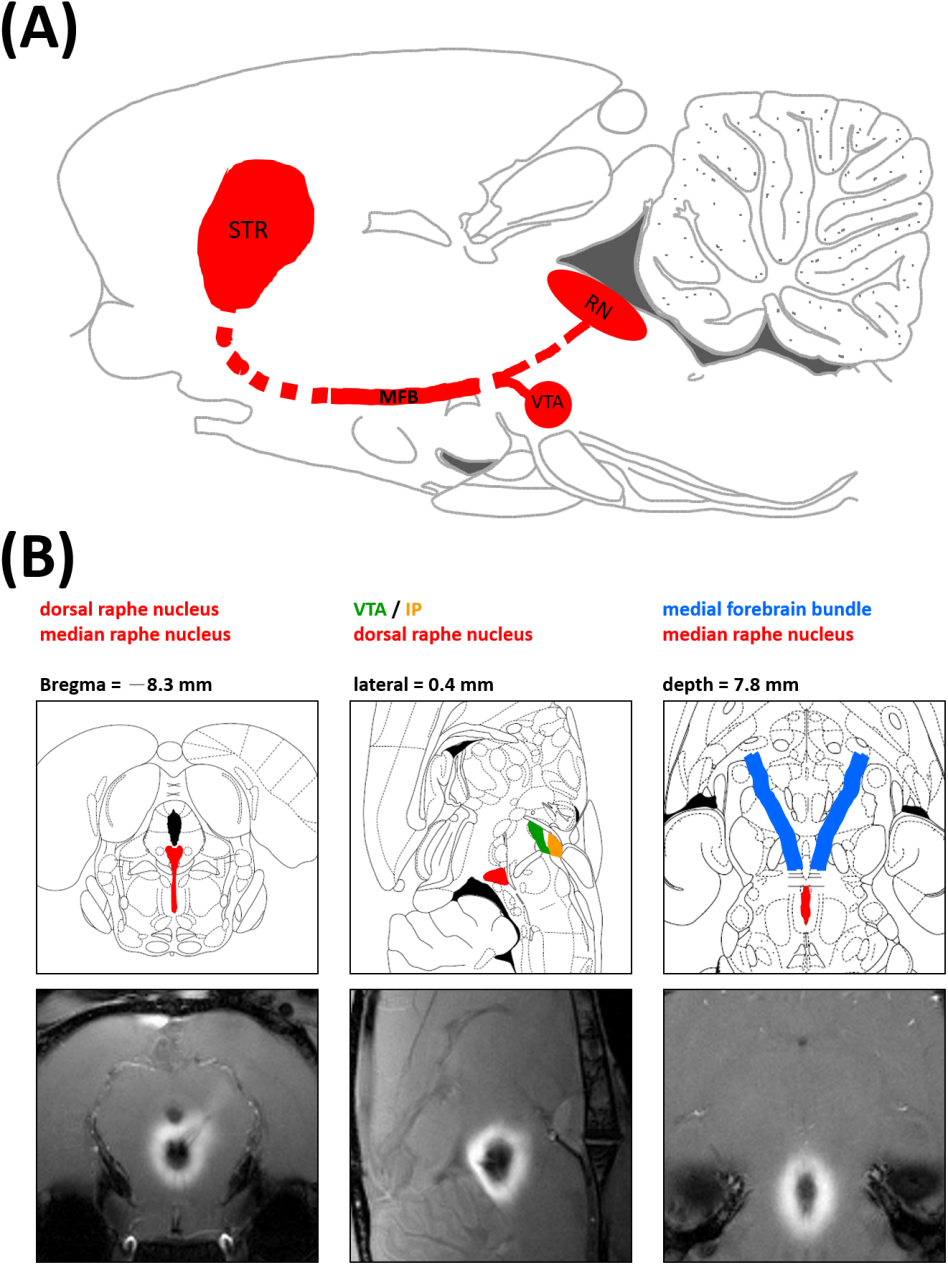
Anatomical interrelationships of the assessed regions. (A) Sagittal view of the midbrain raphe nucleus (RN) and its downstream VTA/IP, MFB, and striatum. (B) Mn^2+^ injection site on the rat brain atlas and on MEMRI images.

After image acquisition, data were analyzed using MRVision software version 1.6.6 (MRVision, Winchester, MA, USA). The T1WIs of each region of interest (ROI) were defined as a series. The T1WIs of each series were manually realigned to the same orientation before processing. ROI placement was performed by two experienced imaging analysts who were blind to the states of the rats under investigation. ROIs were drawn for the VTA, MFB, and striatum based on the Mn^2+^-enhanced T1WIs at different time points, because the period of optimal signal enhancement differed between different regions. The ROIs were manually drawn according to the rat stereotactic atlas as well as the spatial extent of signal enhancement. The VTA and MFB were defined based on the sagittal- and coronal-view T1WIs obtained 8 h post-Mn^2+^ infusion, and the same ROI was then retrospectively applied to all images over all three time intervals. At 38 h after the Mn^2+^ infusion, the striatum was defined based on the axial-view T1WIs obtained 38 h after the Mn^2+^ infusion. The ROI of the striatum was drawn on the slice showed the largest cross section of the structure. Fig 2 illustrates the anatomical positions of the assessed regions: the VTA/interpeduncular nucleus (IP) and the DRN mainly projecting to the striatum via the MFB. Owing to the limitations of MEMRI, the VTA and the IP were structurally indistinguishable and treated as one region for data processing purposes.

Individual changes in relative SI (rSI) were calculated as (SI_nucleus or tract_-SI_control_)/SI_control_, and plotted as a function of time. The muscle was chosen as the control ROI since measurements of SNR in the muscle performed in all rats showed no significant signal changes before and after Mn^2+^ injection (data not shown). The time-rSI curve was fitted with a sigmoid function according to Van der Linden [19,22,26], and given by

rSI=rSImax{1+exp[−(t−T12max)n]} (1)

where rSI_max_ indicates maximal rSI, reflecting the highest Mn^2+^ concentration reached in brain tissue, while the *n* coefficient describes the shape of sigmoid curve and is considered to be a measure of the complexity of the process involved in the uptake, transport, and accumulation of Mn^2+^ in the neuronal pathways. T_1/2max_ is the time needed to reach 50% of the rSI_max_ while *t* is the time after Mn^2+^ injection. The fitting was performed by applying a non-linear regression method using MATLAB software (MathWorks, Natick, MA, USA). To construct the color-coded maps, pixels in the ROIs were extracted and directly converted from gray scale to the color map. The pseudocolored SI maps were then overlaid on the original T1WIs, for visualization using the Amira software (Template Graphics Software, San Diego, CA, USA).

### Immunohistological staining

The animals were sacrificed the day after the end of the MEMRI experiments. The rats were deeply anesthetized with chloral hydrate, subsequently perfused with normal saline and then 4% paraformaldehyde (Sigma-Aldrich) for fixation. The rat brains were then removed, post-fixed in 4% paraformaldehyde, and cryoprotected in phosphate-buffered saline (PBS) containing 20% and 30% sucrose. Tissue blocks were sliced (Leica CM 3050; Leico Microsystem, Taipei, Taiwan) into 5-μm coronal plane sections, which were rinsed with PBS, treated with 1% H_2_O_2_ (Calbiochem, Torrey Pines, CA, USA) in PBS for 0.5 h, incubated in blocking solution [0.5% Triton X-100 (Sigma-Aldrich) and 3% normal goat serum (Vector, Burlingame, CA, USA) in PBS], incubated with rabbit anti-SERT antibodies (1:2000 dilution; Millipore Corporation, Billerica, MA, USA) at 4°C overnight, rinsed with PBS, incubated with goat anti-rabbit biotinylated IgG (1:200; Vector, Burlingame, CA, USA) for 1 h, incubated with avidin-biotin complex (1:200; Vectastain ABC kit; Vector) for 1 h, incubated with 0.05% 3,3-diaminobenzidine (Sigma-Aldrich) for 2.5 min, washed 3 times with PBS, and finally mounted on gelatin-coated glass slides (Thermo Fisher Scientific, Waltham, MA, USA) for examination.

The optical density (OD) measurements of SERT fibers in each brain region were performed according to our previous reports [28,29]. In each brain region, three immunohistological staining sections were selected at a one-section interval from six consecutive sections for quantification. The photographs were acquired using a color CCD camera coupled to a microscope (MICROPHOT-FXA, Nikon, Tokyo, Japan). These photographs were then converted into an 8-bit gray scale (0–255 gray levels) for analysis. Image analysis software (Image-Pro Plus v. 6.0, Media Cybernetics, Inc., Bethesda, MD) was employed to determine the OD values of SERT immunoreactivity. The OD ratio of the target region relative to the reference region (corpus callosum) that is devoid of SERT was defined as (OD of target region–OD of corpus callosum) / OD of corpus callosum.

### Statistical analysis

All numerical data are presented as mean and standard-error of the mean (SEM) values. The first significant increases in Mn^2+^-induced rSI were determined in the selected ROIs of the controls or MDMA-treated rats. Repeated measure analyses of variance (ANOVAs) followed by Fisher’s post-hoc tests were used to carry out the above comparison. Between-group comparisons of rSIs in the ROIs were performed using Student’s *t-*test. The time-rSI curve was fitted to a sigmoid function performed as previously reported. ANOVA (*p* value) and a regression analysis (coefficient of determination, *R* ^2^) were used to examine the degree of fit of each fitted curve. The parameters derived from curve fitting (the maximum SI [SI_max_] and *n*) and the relative OD ratio from immunohistological staining images in the striatum were compared between the MDMA and control groups using Student’ *t-* test. All levels of significance were set at *p<0.05 or **p< 0.01.

## Results

**Fig 3:**
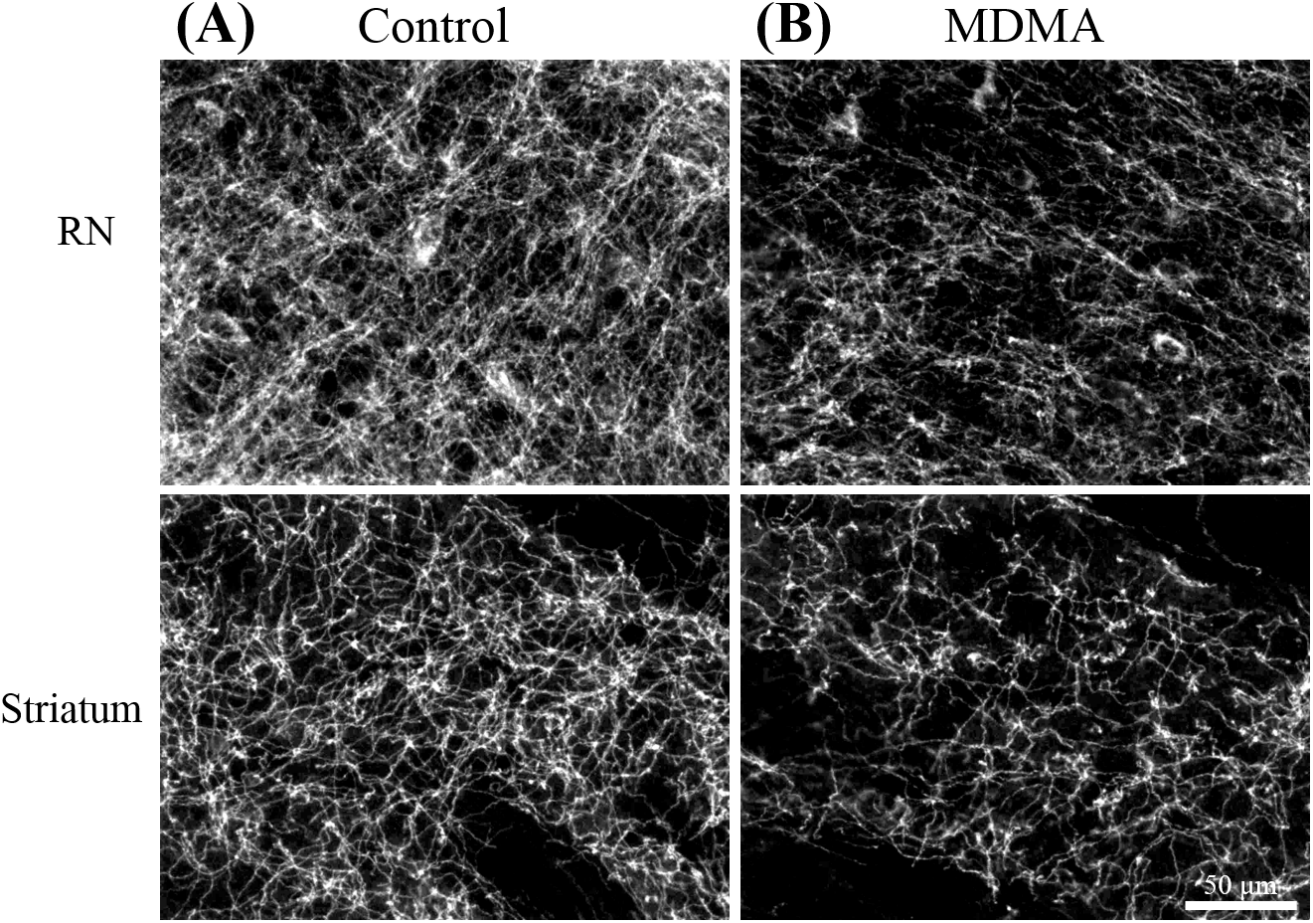
SERT immunohistochemical staining of rat brain sections. Immunohistochemical staining of (A) a control rat and (B) a MDMA-treated rat confirmed the degeneration of serotonergic nerve terminals in the midbrain RN and striatum in the MDMA-treated rat, as shown by reduced SERT expression. Scale bar = 50 μm.

**Fig 4:**
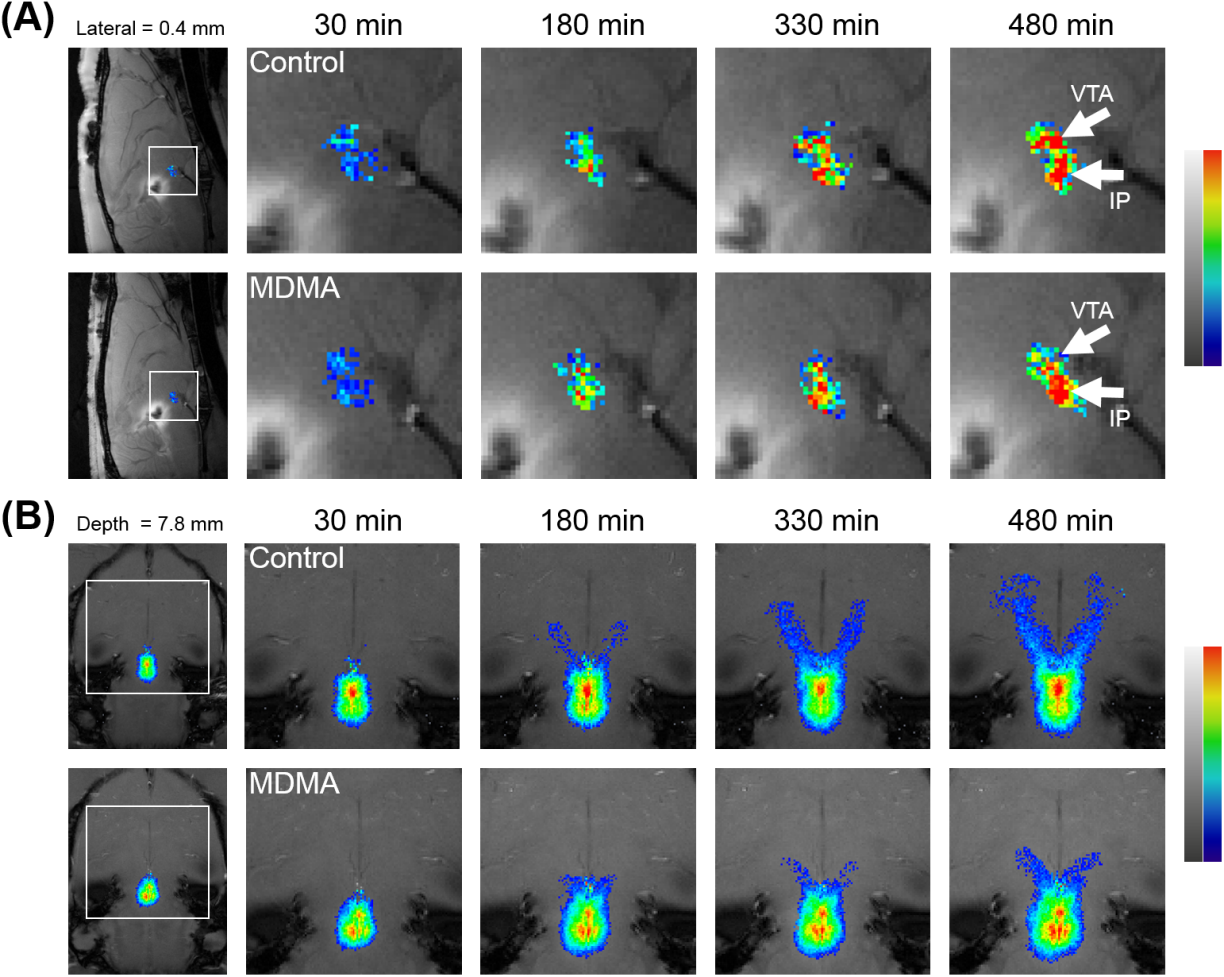
Dynamic MEMRI reveals the temporal and spatial evolution of Mn^2+^-induced signal enhancements. The Mn^2+^-induced signal enhancements along (A) the VTA/IP and (B) the MFB pathway after injecting Mn^2+^ into the midbrain RN. The series of T1WIs shows the kinetics of Mn^2+^ uptake in the midbrain RN, accumulation in the VTA/IP, and transport along the MFB pathway.

As shown in Fig 3, the immunohistological staining revealed that in the density of SERT-positive nerve fibers at the raphe nucleus and the striatum had decreased significantly on the 11^th^ day after the last MDMA treatment, with no significant change evident in the control group. This result is consistent with previous reports of the neurotoxic effects of MDMA on serotonergic nerves [6,14,30]. Both groups showed a gradual, progressive accumulation of Mn^2+^ in the VTA/IP and along the MFB tract, as demonstrated by increased SIs on dynamic MEMRI. Fig 4 shows representative MEMRI scans of MDMA- and saline-treated rats demonstrating selective accumulation of Mn^2+^ in the VTA/IP (Fig 4A), as well as the evolution of Mn^2+^-induced signal enhancement along the MFB (Fig 4B).

**Fig 5:**
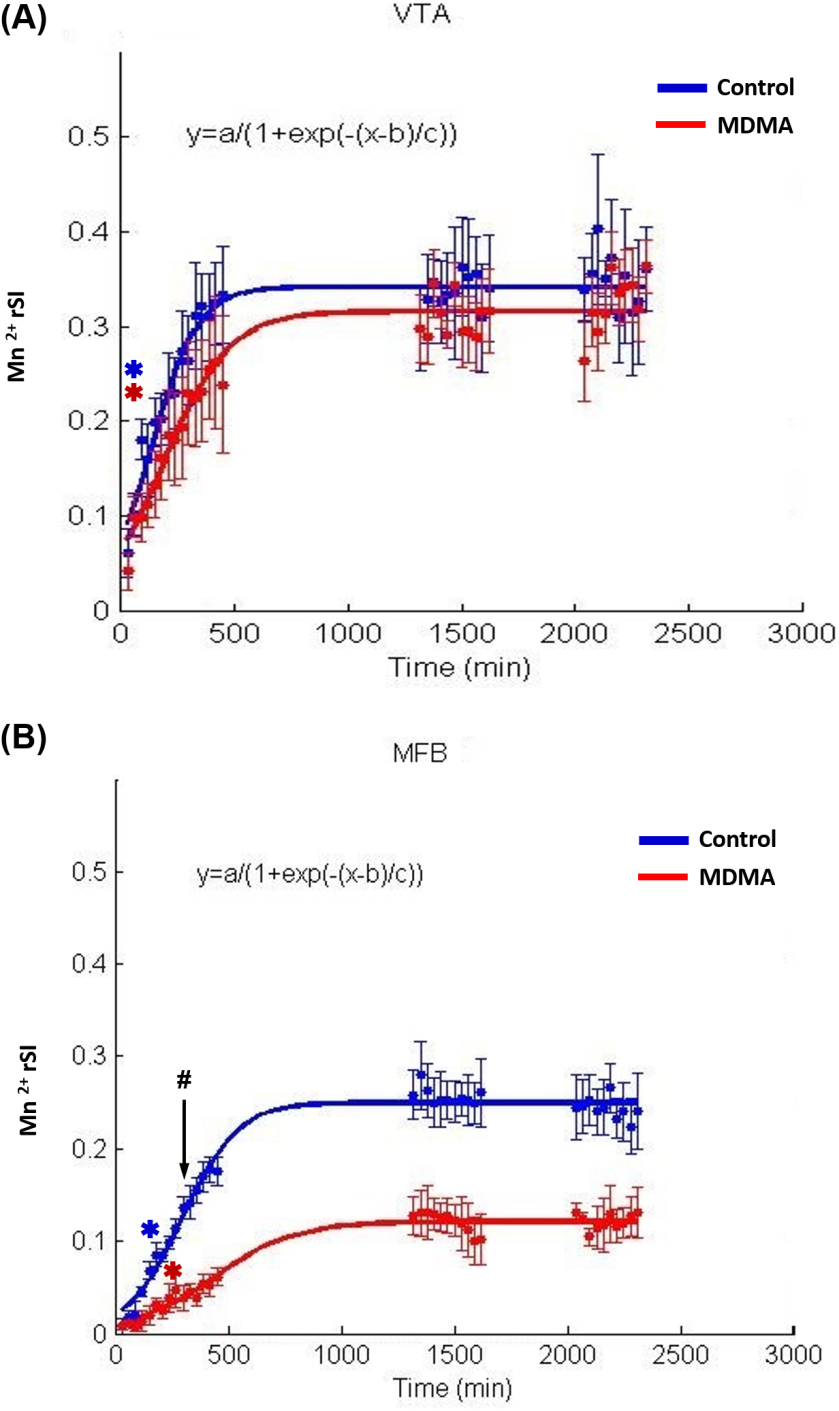
Time course of Mn^2+^-induced rSI reveals the effects of MDMA neurotoxicity. The midbrain RN downstream including (A) the direct target nuclei (VTA/IP) and (B) the MFB pathway. The temporal evolution of the averaged Mn^2+^ rSI in the VTA/IP and the MFB pathway of each group is quantitatively expressed as a function of time, and the data are fitted with sigmoid curves (VTA/IP-MDMA: *R* ^2^ = 0.7359, *p*<0.0001; VTA/IP-control: *R* ^2^ = 0.7990, *p*<0.0001; MFB-MDMA: *R* ^2^ = 0.6221, *p*<0.001; MFB-control: *R* ^2^ = 0.9200, *p*<0.0001). The Mn^2+^-induced rSIs are given as mean and S.E.M. values. Blue and red spots correspond to Mn^2+^-induced rSIs measured from the ROIs in control and MDMA-treated rats, respectively (the corresponding lines are the fitted sigmoid curves). Asterisks indicate the first significant increase in the Mn^2+^-induced rSI, determined by comparing new images with images acquired 40 min after injecting Mn^2+^ (* *p*<0.05). In control rats, the first significant increase in Mn^2+^-induced rSI occurred at 60 min in the VTA/IP and at 120 min in the MFB. In MDMA-treated rats, the first significant increase in Mn^2+^-induced rSI occurred at 60 min in the VTA/IP and at 180 min in the MFB. The rSI of the MFB was significantly lower in the MDMA group than in the control group after 210 min (# *p*<0.05).

Fig 5A and 5B show the time courses of Mn^2+^ evolution in the VTA/IP (Fig 5A) and the MFB, respectively. Increases in the Mn^2+^ rSI were observed over the 2280-min monitoring period in both the MDMA and control groups following Mn^2+^ injection in each ROI, as indicated by repeated-measure ANOVAs. The temporal evolutions of the averaged Mn^2+^ rSI of each group in the VTA/IP and the MFB pathway are quantitatively expressed in the figure as a function of time and are fitted with sigmoid curves. A significant delay in Mn^2+^-induced signal enhancement along the MFB in the MDMA group could be clearly visualized. The Mn^2+^-induced rSI increased in each ROI over the 480-min monitoring period in both groups following Mn^2+^ injection, as indicated by repeated-measure ANOVAs. Fisher’s post-hoc analysis revealed that the first significant increase in Mn^2+^-induced rSI occurred in control rats at 60 min in the VTA/IP [*F*(14,56) = 15.899, *p*<0.003] and at 120 min in the MFB [*F*(14,56) = 45.983, *p*<0.002]. In MDMA-treated rats, the first significant increase in the Mn^2+^-induced rSI occurred at 60 min in the VTA/IP [*F*(14,56) = 11.145, *p*< 0.049] and at 180 min in the MFB [*F*(14,56) = 6.586, *p*<0.024]. The rSI in the MFB was significantly lower in the MDMA group than in the control group after 210 min (*t-*test, *p* < 0.05). However, no between-group significant difference was found in the VTA/IP up to the end of the experiment.

The sigmoid equation (as mentioned above) was applied to further characterize the kinetics of Mn^2+^-induced rSI changes in the VTA/IP and MFB. ANOVAs and regression analysis indicated that the sigmoid curves provided a good model to the collected data (VTA/IP: all *R* ^2^>0.5653, all *p*<0.0001; MFB: all *R* ^2^>0.9104, all *p*<0.0001). Comparison of SI_max_ and the *n* coefficient obtained from each fitted curve between the MDMA and control groups using Student’s *t-*test (Table 1) revealed significant decreases in SI_max_ and *n* in the MFB (*p*<0.05) and a nonsignificant trend toward lower SI_max_ and *n* values in the VTA/IP in the MDMA group compared to the control group.

**Table 1:** rSI_max_ and *n* coefficient values in VTA/IP and MFB of control and MDMA group, as the derived by fitting of time-rSI curve with sigmoid equation.

	VTA/IP	MFB
SI_max_		
Control	0.31±0.050	0.25±0.023
MDMA	0.32±0.021	0.08±0.016*
*n*		
Control	66.56±15.46	127.18±20.28
MDMA	184.71±70.31	67.04±20.48*

* *p* < 0.05

**Fig 6:**
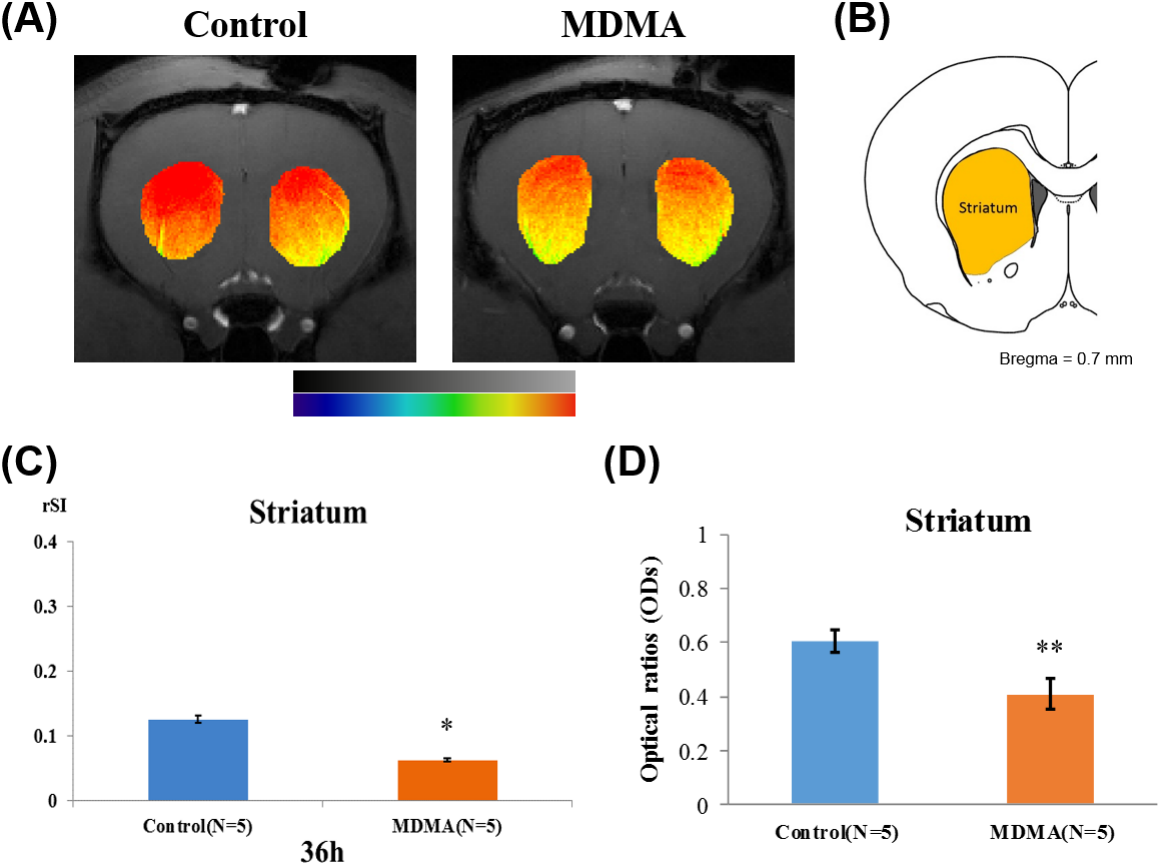
MDMA-induced axotomy of serotonergic neurons in the striatum. (A) MEMRI showing differences in Mn^2+^ distribution in the striatum between control and MDMA-treated rats at 38 h after injecting Mn^2+^. Mn^2+^-induced signal enhancements were decreased after MDMA treatment. The orange-colored region depicts the area of the striatum, as indicated on the atlas in (B). (C) A quantitative analysis confirmed that Mn^2+^-induced rSIs in the striatum differed statistically between control and MDMA-treated rats. The increase in Mn^2+^-induced rSI in MDMA-treated rats was reduced significantly at 34–38 h after injection, compared with controls (*p<0.05). (D) Optical density (OD) ratios of SERT immunoreactivity in the striatum of rat brains from both groups on day 16 after saline or MDMA treatment. Data are presented as mean and S.D. values (** *p*<0.01). There was a statistically significant reduction in the OD ratio after MDMA treatment.

The striatum Mn^2+^-induced rSI changes were assessed at 38 h after injecting Mn^2+^ into the raphe nucleus. Mn^2+^-induced T1WI hyperintensity was observed in the striata of both treatment groups. However, it is clear from Fig 6A that, the enhancement in the striatum was smaller in MDMA-treated rats than in control rats. Fig 6B is a corresponding image taken from the rat brain atlas showing the anatomical location of the striatum. Student’s *t-*test (Fig 6C) indicated that the Mn^2+^-induced rSI in the striatum was significantly smaller in the MDMA-treated group (*p*<0.05).

Immunohistological staining of SERT was carried out at the end of the MEMRI experiment to confirm the MDMA neurotoxicity in the serotonergic terminals in the striatum. Fig 2 depicts two examples of the immunohistological studies in rat brains. SERT-positive fibers were found at the raphe nucleus and striatum in control rat brains, and SERT immunoreactivity was clearly reduced in these regions on the 16^th^ day after MDMA treatment in MDMA-treated rats relative to the control group. The SERT OD ratios were significantly reduced in the striatum on the 16^th^ day after MDMA treatment (relative to controls, *p*<0.01; Fig 6C), that was in line with the decreased Mn^2+^ enhancement observed in this region.

## Discussion

The current MEMRI study successfully employed Mn^2+^-induced enhancement to visualize the disruption of rat raphe-striatal projections after repeated MDMA exposure. A decreased Mn^2+^ accumulation in the striatum was evidenced by the reduced Mn^2+^-induced rSI. Quantitative analysis showed that the kinetic parameters in the MFB differed significantly between MDMA-exposed rats and controls. The MEMRI-identified functional changes are highly consistent with findings from previous histological studies [6,7]. To the best of our knowledge, the present study is the first *in vivo* investigation to provide direct evidence of MDMA-induced axotomy of the long projecting raphe-striatal pathway in an animal model.

Worldwide MDMA abuse since the 1980s has prompted numerous human and animal studies investigating the pharmacokinetics and pharmacodynamics of MDMA [31]. Injecting rats subcutaneously with MDMA at doses of 2–10 mg/kg was found to result in maximal plasma MDMA concentrations after 0.6 and 1.1 h with an elimination half-life of between 1.1 and 2h [32,33]. The injected plasma MDMA is subcutaneously slowly transformed into HHMA (3,4-Dihydroxymethamphetamine) and HMMA (4-Hydroxy-3-methoxymeth- amphetamine) as well as MDA (3,4-Methylenedioxyamphetamine) by hepatic metabolism [1,31,34]. The concentrations for these metabolites peak after about 7 h [32,33]. It is known that MDMA overdose results in a constellation of changes that include muscle rigidity, hypertension, hyperthermia and serotonin syndrome [35]. However, MDMA and its metabolites may have different pharmacodynamic consequences. Recent studies found that the plasma MDMA, MDA and HHMA concentrations were correlated with serotonin syndrome, core body temperature and cardiovascular changes, respectively [33,36]. We previously administered MDMA in a different dose regimen (10 mg/kg, s.c., twice daily for four consecutive days) to study the protective effect of fluoxetine against MDMA neurotoxicity in rats [28]. However, the existing literature in this field suggested that experiments on MDMA neurotoxicity should use the lesser MDMA dose that are more compatible with the dose associated with recreational use of this drug in humans. The current study administered MDMA at a lower dose (5 mg/kg, s.c., once daily for six consecutive days), which successfully induced neurotoxicity in the serotonin system as evidenced by reduced SERT in the rat brains (Fig 3). The reductions of SERT in the striatum in the MDMA group paralleled the disruption of the raphe-striatal projection found in MEMRI. It is, therefore, reasonable to infer that MDMA is toxic to the serotonergic terminals, whereby the axotomy effect contributes to the disrupted axonal transport of Mn^2+^ to the projected brain area.

The mechanism of MDMA neurotoxicity is incompletely understood and so remains an actively researched topic. Several factors have been suggested to be involved in MDMA neurotoxicity, including the formation of toxic metabolites, hyperthermia, mitochondrion dysfunction, and increased oxidative stress [37–39]. Neurotoxicity mediated by oxidative stress seems to be the most plausible hypothesis. It has been shown that MDMA induces the acute release of serotonin and dopamine neurotransmitters [40], as well as increased dopamine uptake at the serotonergic axon terminals via SERT. The subsequent deamination of the abnormally high level of dopamine may induce the production of monoamine oxidase B, a potential source of hydrogen peroxide [37,41]. This explains the selective MDMA-induced destruction of serotonergic fibers although it is unclear why the dopaminergic neurons are spared. This proposed mechanism was supported by our previous *in vivo* rodent study using animal positron emission tomography that demonstrated a protective effect of fluoxetine (a selective serotonin-reuptake inhibitor) against MDMA serotonergic neurotoxicity induced by MDMA [28]. It is further supported by the current *in vivo* MEMRI study demonstrating that the serotonergic neurotoxicity of MDMA is related to terminal axotomy. This series of *in vivo* studies using neuroimaging techniques adds to the growing literature on the serotonergic neurotoxicity of MDMA and clarifies the pharmacopathogenic role of this serotonergic neurotoxicity in living brains.

The present study found that Mn^2+^-induced signal enhancement in the striatum followed a ventrodorsal gradient (Fig 6) both in the control and MDMA rats, with the enhancement being greater in the dorsal striatum. At first glance, this pattern seems to contradict previous findings that the midbrain raphe system preferentially innervates the ventral striatum, although DRN projections have a wide topographical distribution in the striatum and also innervate the dorsal striatum [16]. However, these imaging data need to be more profound interpreted carefully. We attribute this observation mainly to a surface-coil artifact, whereby voxels located further from the surface coil placed above the rat's head may be affected by signal attenuation. Nevertheless, disruption of the raphe-striatal pathway was evident in our study, although the functional importance of this projection remains unclear.

The stimulant and rewarding properties of MDMA on cognition are thought to arise from its ability to activate the mesocorticolimbic dopamine system [42], which originates from the VTA and projects to the nucleus accumbens. Serotonin has been implicated in this MDMA reward-related neurocircuitry via complex interactions between serotonin and gamma-aminobutyric acid (GABA) [43]. Activation of the 5HT_1A_ and 5HT_1B_ receptors in the VTA results in dopamine efflux in the nucleus accumbens, possibly through inhibition of the GABAergic neurons in the VTA and consequently disinhibition of the dopaminergic neurons [42,44,45]. In contrast, activation of the 5HT_2C_ receptors on GABAergic neurons in the VTA inhibits dopamine release [43]. Therefore, the excessive release of serotonin resulting from the acute administration of MDMA will promote the effect of MDMA on mesolimbic dopamine release as well as antagonize its effects by the activation of two groups of serotonin receptors in the VTA. However, the acute effects of MDMA on dopamine release are modest compared to those of other amphetamine derivatives, suggesting that the overall effect of serotonin is generally inhibitory [42]. In line with this idea, the results of an electrophysiological experiment involving normal rats suggest that the DRN which is the main source of serotonergic afferents to the VTA exerts an inhibitory effect on VTA dopaminergic neurons [46].

Chronic exposure to MDMA at high doses has been shown to increase the responsiveness of the mesolimbic dopamine system [42]. It is reasonable to hypothesize that this enhancement results from the diminished inhibitory effect of serotonergic input in the VTA, since long-term MDMA exposure has been shown to damage the DRN axons that project throughout the brain. Nevertheless, the present study found no statistically significant impairment of Mn^2+^-enhancement kinetics in the VTA after repeated MDMA exposure, although a trend toward less enhancements was apparent. There are at least three feasible explanations for this phenomenon. First, given that the VTA is located in close proximity to the Mn^2+^ injection site, the amount of Mn^2+^ transported along the myelin sheath of nerve tract via passive diffusion may be significant and independent of axonal integrity. Second, the sample in the present study may have been too small to allow the identification of all reach statistically significant difference. Third, the small size of this brain structure may lead to significant partial volume effect, as well as large variations when defining the ROI.

A major drawback of using Mn^2+^ as a contrast agent is its cellular toxicity. High concentrations of Mn^2+^ may cause acute cardiovascular collapse as well as neurodegenerative change in the central nervous system. In the current study, the Mn^2+^ solution was delivered at a low concentration (0.1 M) and a high volume (150 nL) and no abnormality was observed after Mn^2+^ injection. The concentration of Mn^2+^ used in the current study was lower than those used in previous MEMRI studies. Hsu *et al*. [19] used 5 nL of 4 M Mn^2+^ to demonstrate disruption in the multisynaptic pathway as a consequence of repeated methamphetamine exposure. Li *et al*. [47] used 100 nL of 0.2 M Mn^2+^ to demonstrate the presence of neuronal projections from the VTA to forebrain structures in rat, and Lehallier *et al*. [48] used 3000 nL of a 0.1 M Mn^2+^ solution in functional imaging to map rat central olfactory structures with odors conveying different biological messages.

The present work implemented an ROI-based approach to the analyze MEMRI data. While this approach has certain benefits, it also suffers from several drawbacks, most notably, the user bias in defining the ROI. This was complicated by the difficulty of readily differentiating some of the brain structures in the Mn^2+^ enhanced images. Also, the relative low resolution of MEMRI may result in significant variability in ROI definitions, especially in the smaller brain structures. This factor may result in the measurements being affected by various partial volume effects. In addition, the ROI-based method requires *a priori* anatomical hypotheses, thereby precluding a systematic full brain analysis. Notably, the use of a voxel-based analysis method [49] could resolve some of these issues, since it essentially permits voxel-wise statistical comparisons to be made between before and after Mn^2+^ injection scans.

One limitation of the present study is the inhomogeneous SI distribution across the image due to the use of a surface coil. The sensitivity of the surface coil reduces with depth; and hence the SI is higher in the region closer to the coil. Although this SI nonuniformity can be corrected through normalization of the image intensity to the signal profile obtained from water phantom, this post-processing technique suffers from two main problems. First, the coil sensitivity varies with certain parameters of the imaging setup, including the shape, size and local tissue architecture of the scanning subject. Moreover, *a priori* knowledge of the coil sensitivity obtained from measurements of phantom signal profiles is usually not sufficiently accurate [50]. Second, although this technique may result in the SI being more homogeneous across the corrected image, the actual localized signal-to-noise ratio remains unchanged. In contrast, the noise in the phantom signal profile may propagate into the final corrected image and degrade the image quality [50].

## Conclusions

In summary, the current study has revealed dysfunction of the raphe-striatal long projecting tract after repeated systemic administration of MDMA in rats, which has not been directly visualized *in vivo* previously. The obtained results demonstrated the potential use of MEMRI in system neuroscience research, also paving the way for future studies in diverse fields.
